# Resequencing at ≥40-Fold Depth of the Parental Genomes of a *Solanum lycopersicum* × *S. pimpinellifolium* Recombinant Inbred Line Population and Characterization of Frame-Shift InDels That Are Highly Likely to Perturb Protein Function

**DOI:** 10.1534/g3.114.016121

**Published:** 2015-03-24

**Authors:** Zoltan Kevei, Robert C. King, Fady Mohareb, Martin J. Sergeant, Sajjad Z. Awan, Andrew J. Thompson

**Affiliations:** * Cranfield Soil and AgriFood Institute, School of Energy, Environment and Agrifood, Cranfield University, Cranfield, Bedfordshire, MK43 0AL, United Kingdom; † Rothamsted Research, Harpenden, Hertfordshire, AL5 2JQ, United Kingdom; ‡ School of Life Sciences, University of Warwick, Gibbet Hill Campus, Coventry, CV4 7AL, United Kingdom

**Keywords:** *Solanum lycopersicum*, *S. pimpinellifolium*, SL2.50, SNP, InDel, introgression, large effect polymorphisms, recombinant inbred lines

## Abstract

A recombinant in-bred line population derived from a cross between *Solanum lycopersicum* var. *cerasiforme* (E9) and *S. pimpinellifolium* (L5) has been used extensively to discover quantitative trait loci (QTL), including those that act via rootstock genotype, however, high-resolution single-nucleotide polymorphism genotyping data for this population are not yet publically available. Next-generation resequencing of parental lines allows the vast majority of polymorphisms to be characterized and used to progress from QTL to causative gene. We sequenced E9 and L5 genomes to 40- and 44-fold depth, respectively, and reads were mapped to the reference Heinz 1706 genome. In L5 there were three clear regions on chromosome 1, chromosome 4, and chromosome 8 with increased rates of polymorphism. Two other regions were highly polymorphic when we compared Heinz 1706 with both E9 and L5 on chromosome 1 and chromosome 10, suggesting that the reference sequence contains a divergent introgression in these locations. We also identified a region on chromosome 4 consistent with an introgression from *S. pimpinellifolium* into Heinz 1706. A large dataset of polymorphisms for the use in fine-mapping QTL in a specific tomato recombinant in-bred line population was created, including a high density of InDels validated as simple size-based polymerase chain reaction markers. By careful filtering and interpreting the SnpEff prediction tool, we have created a list of genes that are predicted to have highly perturbed protein functions in the E9 and L5 parental lines.

Tomato is the most important noncereal crop species after potato. It has simple diploid genetics and a genome of approximately 900 Mb, which has been widely studied. The reference genome (~820 Mb) of *Solanum lycopersicum* (Heinz 1706) is publically available (Tomato Genome Consortium 2012) and has been used extensively for the identification of single-nucleotide polymorphisms (SNPs) in range of cultivars and related species (Sim *et al.* 2012a,b) and in genome resequencing projects (Viquez-Zamora *et al.* 2013). SNP markers of expressed sequence tags were used to create diverse tomato linkage maps and for comparative analyses among cultivars (Shirasawa *et al.* 2010), another study of 40 tomato lines revealed many SNPs that may affect gene function (Hirakawa *et al.* 2013). Genotyping using SNP arrays confirmed that SNPs from wild-species had been introgressed into domesticated cultivars (Viquez-Zamora *et al.* 2013). Shirasawa *et al.* (2013) showed that detailed SNP analysis of six tomato lines coupled with genome wide association studies can directly associate SNP data with agronomical traits. The resequencing of eight tomato accessions by next-generation sequencing demonstrated the presence of introgression regions between varieties and revealed intragenic polymorphisms, including SNPs, insertion−deletion mutations (InDels) and copy number variation (Causse *et al.* 2013). Moreover, the available “150 tomato genome re-sequencing project” (100 Tomato Genome Sequencing Consortium *et al.* 2014), which was augmented very recently with SNP data from another 360 accessions (Lin *et al.* 2014), offers a huge selection of resequenced genomes allowing a myriad of comparisons of SNPs and InDels between various tomato landraces, heritage cultivars, and wild relatives.

A tomato recombinant inbred line (RIL) population was developed from a cross between the *S. lycopersicum* var. *cerasiforme* (cherry tomato) line “E9” and the wild species *S. pimpinellifolium* line “L5” (Monforte *et al.* 1997). The *S. lycopersicum* var. *cerasiforme* falls within the same species as cultivated tomato, but possesses considerable morphological diversity and is believed to be an admixture of wild and cultivated tomatoes that has perhaps arisen from reversion of cultivated forms (Peralta *et al.* 2008). *S. pimpinellifolium* is a wild tomato species closely related to *S. lycopersicum* that is self-compatible, and that can be crossed with the domesticated lines without hybrid incompatibility in subsequent generations. Various agronomic observations have highlighted the importance of *S. pimpinellifolium* germplasm in tomato breeding: it has natural resistance against the two-spotted spider mite (Fernández-Muñoz *et al.* 2003), whitefly (Rodríguez-López *et al.* 2011), and late blight (Foolad *et al.* 2008). *S. pimpinellifolium* has been widely used as a parent in QTL studies (Weller *et al.* 1988), including the localization of QTL for fruit shape and size (Tanksley *et al.* 1996; Grandillo *et al.* 1999). As the *S. pimpinellifolium* LA1589 has been recently sequenced (Ware *et al.* 2014) the resultant InDel sequence data were used to calculate the genetic distance between different cherry tomatoes and other *S. pimpinellifolium* lines (Yang *et al.* 2014). The existing 7720 SolCAP SNP markers were used to select a substantial number of introgression lines in the pest resistant *S. pimpinellifolium* TO-937 line (Barrantes *et al.* 2014).

The RIL population generated from the cross of E9 × L5 has been phenotyped with the aim of exploring salinity tolerance and rootstock-specific traits. The microsatellite coverage of the F_6_ RILs from the cross E9 × L5 (P population) were compared to that of F_6_ RILs derived from the cross of E9 with another salt tolerant tomato wild species accession, *S. cheesmanii* (C population) (Villalta *et al.* 2005). The F_7_ RILs of the P and the C populations also were used for comparative analyses and mapping of QTL responsible for salt tolerance in relation to fruit yield (Villalta *et al.* 2007). The physiology of salinity stress was then analyzed in the F_8_ populations via the investigation of QTL for Na^+^ and K^+^ concentrations in stems and leaves (Villalta *et al.* 2008). The effect of rootstock genotype on salt tolerance was analyzed in the F_9_ RILs of the P and C populations, revealing a QTL which had a direct rootstock effect on fruit weight under high salinity conditions (Estan *et al.* 2009). Furthermore, this fruit weight QTL of the P population (gFW9.1) cosegregated with a leaf water-content QTL (g5LW9.1) on chromosome 9 increasing the agronomic importance of these rootstock dependent QTL for breeding (Asins *et al.* 2010). The detailed analyses of one of the above mentioned QTL related to Na^+^ and K^+^ homeostasis in leaves identified two closely linked HKT transporter genes on chr 7 which are good candidates to be causative genes underlying a salt tolerance QTL (Asins *et al.* 2013). The P population has been genotyped for 7720 SolCAP SNP markers (Asins *et al.* 2013), and 278 of these were used to map the region containing the two HKT genes, but the SolCAP genotype scores are not yet publically available.

To allow rapid progress in fine-mapping of QTL discovered in the P population, we have resequenced the genomes of the parental lines E9 and L5 to a depth of 40-fold by mapping reads against the Heinz 1706 reference. Subsequently we have made a detailed analysis of the discovered polymorphisms, focusing on introgressions and mutations that are likely to perturb protein functions.

## Materials and Methods

### Plant material, sequencing, and polymerase chain reaction (PCR)

Total genomic DNA of *S. lycopersicum* var. *cerasiforme* line E9, *S. pimpinellifolium* line L5, P population individuals (F6 generation) and Micro-Tom line was isolated from leaf tissue using DNeasy 96 Plant Kit (QIAGEN). Whole-genome sequencing was carried out by Illumina HiSeq (L5 and E9, one lane each) using 100 bp paired-end reads, or by Illumina Genome Analyzer using 76-bp paired-end reads with an average insert size of 533 bps (Micro-Tom). Specific oligonucleotides were used in standard PCR conditions for the closely linked PCR InDel marker of gFW9.1. 3% agarose gel and 0.5x TBE buffer was used for visualization of PCR amplifications.

### Alignment and variant calling

Burrows-Wheeler aligner (BWA-MEM algorithm; version 0.7.4) was used to align the sequencing reads, with default parameters, to the *S. lycopersicum* reference genome sequence version SL2.50 (Sol Genomics Network). Alignments were converted to sequence alignment map format followed by binary alignment map, sorted and indexed (Samtools version 0.1.19). Picard tools were used to mark duplicate reads post mapping. GATK software tools (version 3.3.0; Broad Institute) were used for InDel realignment and variant calling (HaplotypeCaller), using default settings.

### Annotation and filtering

Resulting “VCF” files were annotated using SnpEff (version 4.0e) (Cingolani *et al.* 2012) with ITAG2.40 annotation (Sol Genomic Network). Filtering was performed using a combination of SAMtools to remove the polymorphisms with coverage of above 100 (Quality Depth < 2 / Fisher Strand > 60 / Mapping Quality < 40 / ReadPosRankSum 8 / Mapping QualityRankSum 12.5), SnpSift (version 4.0e) to remove heterozygous polymorphisms and filter frame shifts (FS), and GATK SelectVariants to filter for InDels or SNPs. Polymorphism physical positions from the zipped and indexed VCF files were compared using vcf-compare from VCFtools (version 0.1.12b).

### Gene expression levels

RNA expression levels were obtained from the RNA-seq data (ftp://ftp.solgenomics.net/genomes/Solanum_lycopersicum/RNA_seq/) of Sol Genomics Network (SL2.40_all_rna_seq.bigwig). *ITAG2.3*/SL2.40 RNA-seq coverage data on genes with relative maximum value <200 (blue color) were classified as having a “nondetectable” gene expression level.

### Data availability

The sequence data were displayed in “Genoverse” (http://www.genoverse.org/) interactive genome browser to visualize and localize SNPs, InDels, and homozygous FS of L5 and E9, and the SNPs of Micro-Tom: http://elvis.misc.cranfield.ac.uk/genoverseSol/examples/tomato/chromosome01.html. Note that the GATK SelectVariants tool provides the E9 and L5 variants with the greatest mapped-read frequency for display on Genoverse: minor variants at a particular locus are not displayed as they are assumed to be due to false mapping rather than genuine heterozygosity in these inbred lines. Variants are color coded on Genoverse to indicate quality score: red indicates the highest quality, green the lowest and orange intermediate. In addition, the Micro-Tom SNPs are available from the Sol Genomics Network genome browser (http://solgenomics.net/gb2/gbrowse/ITAG2.4_genomic/).

## Result and Discussion

### Sequence analyses: SNPs and InDels

For sequence polymorphism discovery, we isolated the genomic DNA from the two parental lines, *S. lycopersicum* var. *cerasiforme* line E9 and *S. pimpinellifolium* line L5. Illumina sequencing resulted in 370 million reads for E9 and 380 million reads for L5. The sequencing reads were mapped to the latest version (SL2.50) of the Heinz 1706 reference tomato genome (Tomato Genome Consortium 2012) with a 40- and 44-fold coverage for E9 and L5, respectively. This depth of coverage is greater than in many other tomato genome comparisons, in which 8- to 20-fold coverage of sequences was used (Causse *et al.* 2013; Shirasawa *et al.* 2013). The larger depth of coverage gives more reliable polymorphic data reducing the impact of low quality reads and the problems of mapping to regions of repetitive DNA. The SnpEff software was used to determine polymorphisms between the parental lines (summarized in Supporting Information, Table S1). In addition, in an earlier study, we had determined SNPs between *S. lycopersicum* cv. Micro-Tom (Scott and Harbaugh 1989), and Heinz 1706 after sequencing the Micro-Tom genome to approximately 10-fold depth of coverage; therefore we included this cultivar as well in the following analysis.

**Figure 1:**
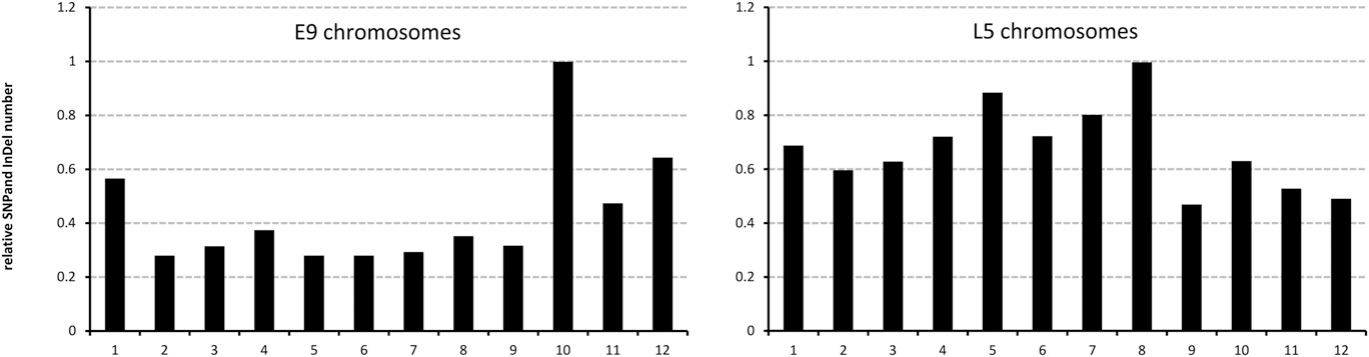
Relative distribution of single-nucleotide polymorphisms (SNPs) and insertion−deletion mutations (InDels) on the E9 and L5 chromosomes. The number of SNPs and InDels per unit length of chromosome are given for each chromosome. For both genomes, values are expressed relative to the chromosome with the maximum linear density of polymorphisms.

In comparison with Heinz 1706, E9 possesses many fewer sequence changes than L5; this is expected because the cherry tomato is a closer relative of the cultivated variant (Hirakawa *et al.* 2013). Approximately 900,000 changes were found in E9 with an average of ∼67,000 per chromosome (~69,000 if the nonassembled chr 0 data are included); this represents approximately one sequence change every 1100 base pairs (bp). Interestingly there is a higher accumulation of sequence changes on chr 10 (Figure 1). With around 150,000 changes, it has triple the number found on chr 4, 5, or 8, which are of a similar length. Furthermore, chr 1, which is 1.5-fold longer than chr 10, has considerably fewer sequence alterations in total. In contrast, the particularly high number of changes on chr 1, chr 10, and chr 12 in E9 was not found in other cherry tomato lines where the rate of polymorphisms was highest on chr 4, 5, and 8 (Causse *et al.* 2013). This finding suggests that there are major differences in the genome structures of different accessions, which are all classed as *S. lycopersicum* var. *cerasiforme*. It is proposed that the lines used by Causse *et al.* (2013) are an admixture of *S. lycopersicum* and *S. pimpinellifolium* genomes, whereas the origin of *S. lycopersicum* var. *cerasiforme* line E9 appears to be a different admixture, consistent with multiple events in which reversion of cultivars back to the wild creates many independent forms of *S. lycopersicum* var. *cerasiforme* (Ranc *et al.* 2008).

In the *S. pimpinellifolium* L5 line, more than seven million sequence changes were detected with an average of ~610,000 per chromosome (~590,000 if the nonassembled chr 0 changes are included); this represents one sequence change every 111 base pairs. In general, therefore, the number of polymorphisms between L5 and Heinz 1706 is approximately 10 times greater than the number between E9 and Heinz 1706. In contrast to E9, the sequence changes in L5 were relatively evenly distributed between the 12 chromosomes (Figure 1). The most notable variation was that chr 8 had twice the linear density of polymorphisms as chr 9. It is worth mentioning that chr 5 and chr 8, which are highly divergent L5 chromosomes, also show prominent sequence changes in the cherry-type tomato admixture lines of *S. lycopersicum* and *S. pimpinellifolium* (Causse *et al.* 2013), suggesting that some of the admixture from *S. pimpinellifolium* is present on these two chromosomes (Ranc *et al.* 2008).

### Intrachromosomal SNP and InDel distributions and putative introgression sites

**Figure 2:**
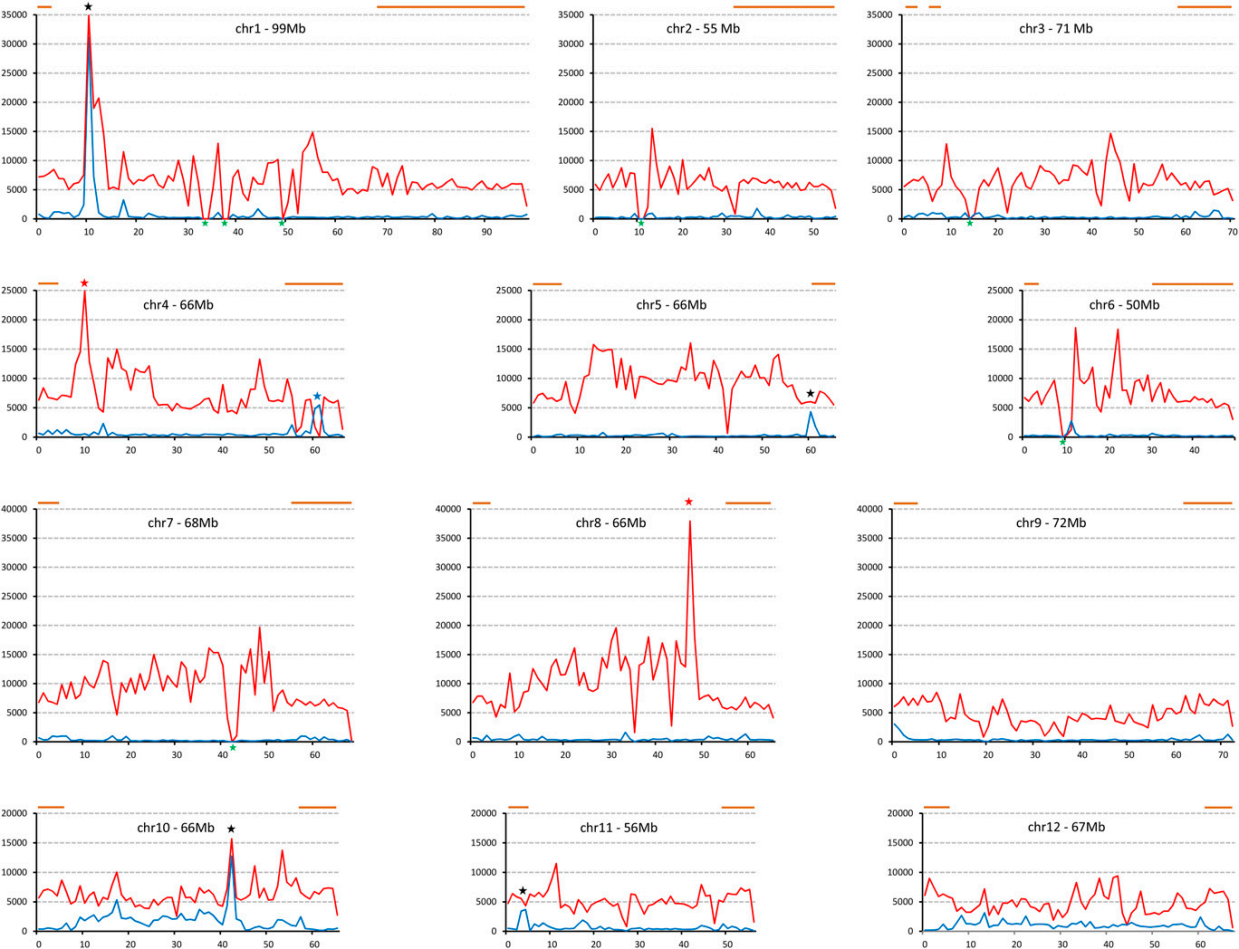
Linear density of single-nucleotide polymorphisms (SNPs) along the chromosomes of E9 and L5. The number of SNPs within each 1-Mb window (Y axis) is plotted against chromosomal positions for each of the 12 chromosomes (X axis). Blue: E9; red: L5. Blue star marks the E9 specific SNP accumulations, red stars stand for L5 specific increase. Black stars stand for similar SNP score in both lines; green stars show the regions with a zero SNP value because of gaps in the SL2.50 reference sequence. Gene rich regions (more than 50 genes in 0.5 Mb) are marked with a brown line.

SNP and InDel distributions on each chromosome were analyzed to reveal the regions which are the most polymorphic to the reference genome. Most chromosomal sections in L5 had between 2000 and 20,000 SNPs per megabase (Mb) (Figure 2). However, there were three chromosomal segments where the number of SNPs was particularly high (Figure 2); chr 1 (9.63−11.56 Mb), chr 4 (9.15−11.60 Mb) (Figure S1) and chr 8 (48.48−48.76 Mb) (Figure S2). Because these peaks are absent from E9 (so that E9 and Heinz 1706 are similar in these regions) the most likely hypothesis is that there were ancestral introgression events that occurred in the progenitor of E9 and Heinz 1706 where the donor species was highly divergent from L5. Alternatively the peaks in L5 may simply reflect different ancient origins of the *S. pimpinellifolium* DNA and consequent variable divergence from Heinz 1706 and E9.

Similar to the situation in L5, there are also chromosomal regions in E9 possessing noticeably more SNPs than the surrounding DNA (Figure 2): chr 5 (60.11−61.24 Mb) (Figure S2) and chr 11 (3.51−4.62 Mb) (Figure S3). In these locations we detected almost as many changes as observed in L5, with approximately 5000 SNPs within 1 Mb. This finding suggests introgression regions that are specific for E9 because the changes are rather different from the SNPs found in L5 or Micro-Tom. In addition to these two E9 regions, there are another two chromosome sections where there were peaks in genetic differences, where approximately more than 10,000 SNPs were found (Figure 2), which was much higher than the typical ∼1000 mutations per Mb in E9. Interestingly, both regions (chr 1; 10.55−11.14 Mb and chr 10; 42.11−42.37 Mb) are also highly polymorphic in L5 (Figure 2, Figure S3, and Figure S4); the similar positions and identities of polymorphisms in these two regions suggests an introgression in Heinz 1706 derived from sources divergent to both L5 and E9. Micro-Tom also shows high polymorphism in the chr 1 region (10.55−11.14 Mb), further supporting the idea of a divergent introgression in Heinz 1706 in this position (Figure S3), whereas in the chr 10 region (42.11−42.37 Mb) Micro-Tom is almost identical to the reference genome, signifying that this probable introgression is present in both Heinz and Micro-Tom (Figure S4). A further possibility is that an introgression occurred in E9 or L5 which was then transferred to the other during breeding or natural hybridizations. A region on chr 4 between 60.32−62.03 Mb shows a unique property in E9: it possesses more SNPs on average than L5, making it the only region in E9 with this property. The high accumulation of changes in E9 and the almost entire absence of SNPs and InDels in L5 strongly indicate an introgression event occurred in Heinz from *S. pimpinellifolium*. Moreover, Micro-Tom possesses highly similar SNPs and InDels to E9 in this region which also supports the introgression of *S. pimpinellifolium* in Heinz (Figure S4). Interestingly, these data seem to differ from the results of another sequencing project where the distal part of chr 4 showed high SNPs between Heinz 1706 and S. *pimpinellifolium* lines (Tomato Genome Consortium 2012). The origin of this particular region is of interest because numerous, potentially disturbed genes are located within it (Figure S4).

The InDels showed similar chromosomal distributions to the SNPs. The obvious difference between them is the lower (5−10 times) amount of InDels in the same region. Similarly to the SNP data, chr 1 (Figure S1) and chr 8 (Figure S2) contain a prominent number of InDels in L5. This is not always true, however, because in the case of chr 4, the InDel number is lower than might be expected considering the SNP linear density. On chr 5 (Figure S2) and chr 11 (Figure S3) of E9, there are also significantly more InDels compared with the SNP density in these regions; however it is not possible to determine whether these alterations are significant or due to variations in the mapping quality in these two cases. The SNP-rich region of E9 on chr 1 and chr 10 (42.11-42.37 Mb) also possess large number of InDels. Moreover, the region chr 4 between 60.32-62.03 Mb (Figure S4) carrying E9 specific SNPs has also elevated number of InDels. Thus, most InDel values reflect the corresponding SNP pattern along the chromosomes.

**Figure 3:**
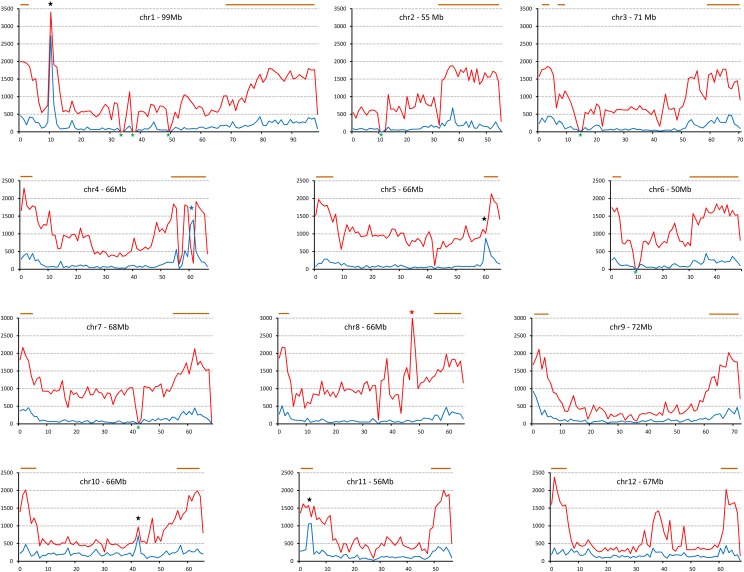
Linear density of insertion−deletion mutations (InDels) along the chromosomes of E9 and L5. The number of InDels within each 1-Mb window (Y axis) is plotted against chromosomal position for each of the 12 chromosomes (X axis). Blue: E9; red: L5. Blue star marks the E9-specific single-nucleotide polymorphisms (SNPs) accumulation, red star stands for L5 specific increase. Black stars stand for high SNP score in both lines; green stars show the regions with a zero InDel value because of gaps in the SL2.50 reference sequence. Gene rich regions (more than 50 genes in 0.5 Mb) are marked with a brown line.

Surprisingly, there is a significant difference in the SNP and InDel distributions at the chromosome scale in both E9 and L5. For SNPs, apart from the above discussed specific regions with high or low SNP accumulations, the rest of the SNPs are relatively evenly distributed along the chromosomes in both lines. In contrast, the InDels have higher density close to the proximal and distal part of the chromosomes (Figure 3). The difference between the SNP (Figure 2) and InDel (Figure 3) accumulation is rather apparent in the case of chromosome 9 and 10. Indeed, the pattern of InDel density follows the accumulation of genes in the same region (Figure 3) (Kobayashi *et al.* 2014). This overlap between genes and InDels suggests that the accumulation and/or selection of InDels is related to the euchromatin, the gene-rich regions, where recombination events may predominantly occur, while the SNP distributions are more evenly distributed between euchromatin and heterochromatin in the tomato genome. These findings are in accordance with the investigations of SNP and InDel distributions in sorghum chromosomes (Evans *et al.* 2013).

Beside the high sequence variations, both E9 and L5 lines possessed regions were the SNPs and InDels were at a low level or completely absent within a 1 Mb window (Figure 2 and Figure 3). These regions correspond to large gaps in the reference genomic sequence. Most likely they overlap with the highly heterochromatic tomato centromeres (Chang *et al.* 2008) containing repetitive sequences which impeded the *de novo* assembly of the reference sequence.

These observations on the SNP and InDel distributions give important information about the potential introgressions and can contribute to our understanding of the QTL variations and their ecological significance in the genetic backgrounds in which they underwent natural selection. The data on the frequency of polymorphisms are important in designing strategies to fine map QTL to identify the genes responsible for influencing the trait.

### InDel frequency and sizes

In addition to the 742,963 SNPs, 93,712 insertions and 55,702 deletions were found in E9. L5 had 432,545 insertions and 380,701 deletions beside the 6,934,608 SNPs. The size of detected insertions and deletions compared to Heinz 1706 were up to ~200−300 bp, even in the case of the more closely related E9. The longest natural deletions found were 212 bp in E9 and 238 bp in L5, whereas the longest insertions were 319 bp (E9) and 326 bp (L5). Compared with the reference genome, E9 has 394 deletions and 345 insertions longer than 50 bp, whereas the equivalent figures for L5 were 3445 deletions and 4106 insertions. This large number of InDels and their relatively even distribution along gene-rich regions facilitates the generation of simply-detectable polymorphic InDel-based molecular markers that can be used for fine genetic mapping of traits in the investigated RIL population. We have used even shorter (between 10-32 bp) InDel-based PCR markers in the process of fine mapping fruit weight QTL gFW9.1 (Estan *et al.* 2009) (Figure S5). As InDels can be found mostly in the gene rich regions (Figure 3) we were interested to analyze the actual coding regions in further detail to see potential gene effects on gene function.

### Resolution of SNPs and InDels in coding sequences

A further aim of this study was to search for SNPs and InDels that would have major effects on the function of gene coding sequences, and that might be expected to confer interesting and significant phenotypes. We observed that E9 and L5 showed very similar distributions between the different types of changes related to either coding or noncoding genomic sequences, but L5 has approximately 10 times more differences in each category. E9 has 41,593, whereas L5 has 410,684 changes in intron sequences. In the exons, E9 had 23,023 SNPs and InDels, whereas L5 had 150,623. The intergenic regions of E9 contain ~800,000 sequence changes while L5 has ~7 million. Thus the ratios of coding to noncoding sequence changes were comparable between the two parent lines: 35 and 45 times more SNPs and InDels were detected in noncoding *vs.* coding regions of genes in E9 and L5, respectively.

We carefully investigated the distinct change types within the coding regions to determine the degree to which they were expected to alter protein structure and function. The category with the highest number of changes is the non-synonymous amino acid (aa) differences where E9 has more than 13,000 while L5 has approximately 83,000 changes (Table S2). These numbers, particularly in the *S. pimpinellifolium* L5 line, are notably high because the number of detected nonsynonymous aa changes was more than the double of the total genes number (~36,000) in the investigated plant genome. Without functional genetic or transgenic studies it is difficult to conclude whether these aa changes result in any alteration in phenotype. However, the study of InDel data (Table S2) to discover more severe protein sequence changes could be valuable in finding genes underlying QTL or monogenic mutations. Frame shift modifications may result in unquestionable protein sequence modification and consequently altered or null functional characteristics.

### Genes influenced by InDels causing FS

**Figure 4:**
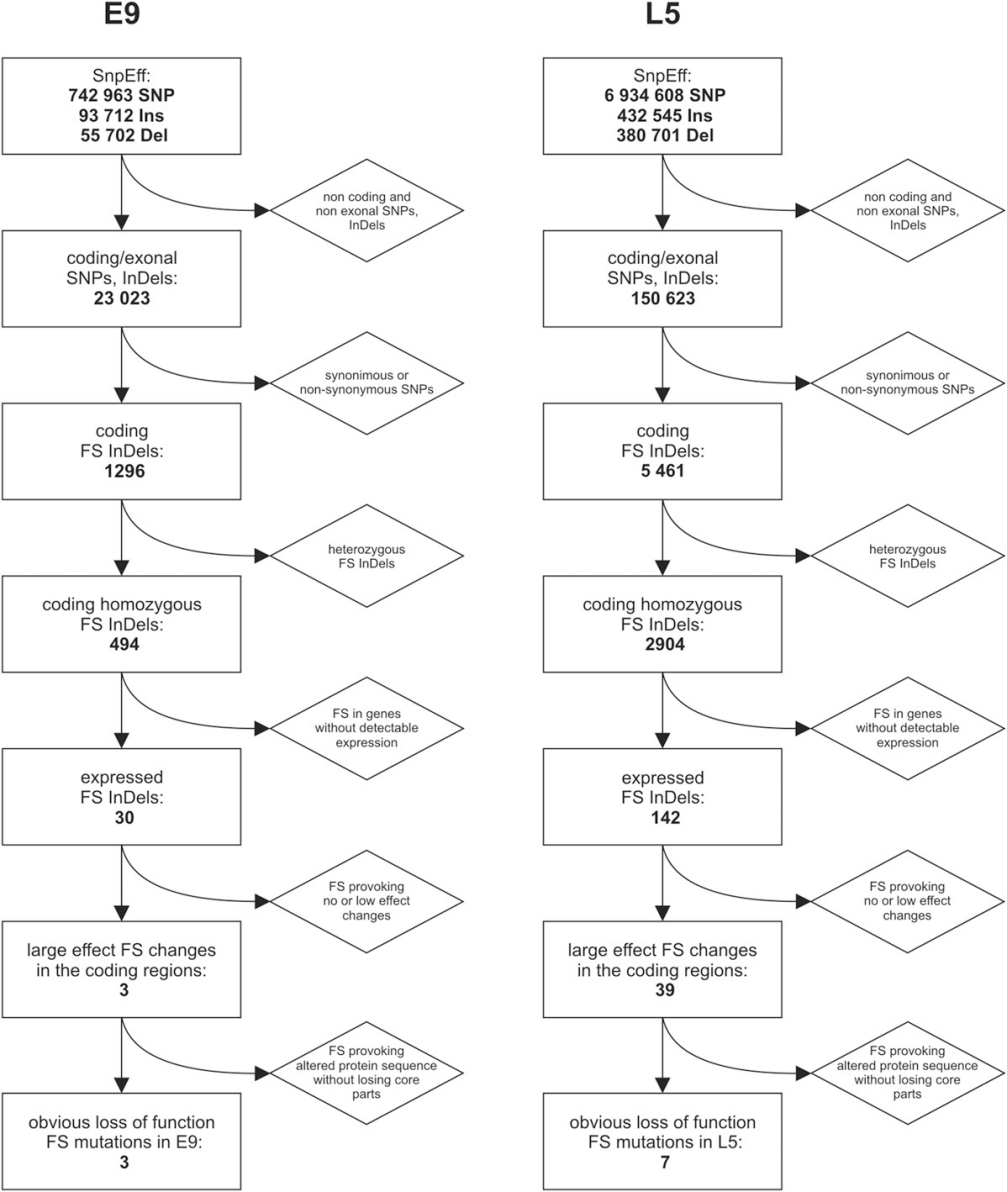
Filtering process for frame shift (FS) mutated genes. The different steps of the flowchart show the process of establishing which FS insertion−deletion mutations (InDels) led to genuine, large effect changes in the protein structure. SNP, single-nucleotide polymorphism.

Using SnpEff software we found 1296 and 5461 FS InDels in E9 and L5, respectively (Table S2). This raises the question of whether it is plausible to have such a large number of disruptive gene changes without rendering a genome unviable. Therefore, we investigated these FS InDels further using a systematic manual analysis protocol to determine which persuasive, large effect gene changes (McNally *et al.* 2009) were most likely to cause functional alterations (Figure 4). First, we eliminated the genes which have heterozygous InDels. Given that the E9 and L5 lines are the results of inbreeding, they must possess very low residual heterozygosity, and potential sequencing and read mapping errors are the most likely origin of heterozygous polymorphisms. Therefore, we chose to study the coding regions possessing only homozygous FS changes. This resulted in 494 genes in E9 (Table S3) and 2904 in L5 (Table S4). Afterward, based on RNA-seq results within existing databases, the genes without detectable RNA expression levels were also filtered out to focus on only genes with experimentally verified transcriptional activity. Of the 494 E9 genes containing FS InDels, only 30 seems to be considerably active, the others are likely nonfunctional or pseudogenes, or possibly expressed in rare conditions, or at a very low level. L5 has 2904 FSs in genes, but only 142 have reported RNA expression level. Next we discovered that several insertions or deletions mark the start or stop codon sites or the exon-intron borders, where the change of one base pair provoked no genuine FS. These are mostly insertions and deletions of a base pair that is identical to its neighbor resulting in no actual FS. These can be considered pseudo FS, detected by an imprecise algorithm in the SnpEff software. If we exclude these pseudo FS, E9 has now only three remaining genes containing FS InDels (Table 1). These three InDels have large and clear effects on the three proteins because they miss entire functional domains (Table S5). Excluding the pseudo FS in L5, reduced the number of FS InDels from 142 to 39 (Table 2), and these 39 mutations were studied more comprehensively. Most seemed to result in a small shortening of the N or C terminal part of the proteins by only a few aa which may presumably not provoke major functional modifications (Table S6), for example in the case of *Solyc01g058160*, *Solyc01g095620* or *Solyc09g082630* genes in L5 (Table 2). By aligning with BLAST the FS InDel-containing L5 protein sequences with available database sequences, it was apparent that some of them were more likely to represent complete, functional L5 genes, and the allelic annotated gene of the reference genome was therefore either functionally compromised, or wrongly predicted during the annotation. For instance, the analysis showed that in some cases the L5 protein structure was more similar to the potato ortholog than to the Heinz 1706 reference allele. This is likely the case for *Solyc01g050040*, *Solyc06g005080*, *Solyc06g084160* and *Solyc11g005420* genes.

**Table 1:** The frame shift altered proteins in E9 compared with reference genome

Chr	Gene Number	Gene Annotation	Sequence and Structural Change in E9
3	Solyc03g115650	Translation initiation factor 5A-1	Missing most part of the protein, including the S1-like RNA recognition motif
12	Solyc12g038920	Serine/threonine-protein kinase 16	Missing most part of the protein, truncated kinase domain
12	Solyc12g100290	Histone-lysine *N*-methyltransferase ASHH3-like	Missing most part of the protein, truncated SET domain

The table explains the effects on protein modification.

**Table 2:** The frame shift altered proteins in L5 compared with reference genome

Chr	Gene Number	Gene Annotation	Sequence and Structural Change in L5
1	Solyc01g005290	SEC14 cytosolic factor protein	Altered C-terminal sequence*^a^*
1	Solyc01g017050	PG1 protein like	Altered C-terminal sequence*^b^*
1	Solyc01g050040	C3HC4-type RING finger protein	Altered N- and C-terminal sequence*^a^*
1	Solyc01g058160	Agenet domain-containing protein	Altered C-terminal sequence
1	**Solyc01g090610**	**Gibberellin 2-beta-dioxygenase 7-like**	Missing most part of the protein including the prolyl 4-hydroxylase a subunit
1	Solyc01g091150	Golgi SNAP receptor complex member 1-2-like	Altered C-terminal sequence*^b^*
1	Solyc01g095620	Hydroquinone glucosyltransferase-like	Altered C-terminal sequence
1	Solyc01g095680	Root primordium defective 1-like	Altered C-terminal sequence
1	Solyc01g103200	Conserved uncharacterized protein	Possessing a longer new protein sequence on C-terminal*^a^*
2	Solyc02g064630	Telomere repeat-binding factor 1-like	Altered C-terminal sequence
3	**Solyc03g098240**	**Glutamate decarboxylase isoform 1**	Missing most part of the protein, the glutamate decarboxylase domain is truncated
3	Solyc03g111720	Peptide methionine sulfoxide reductase	Altered C-terminal sequence
3	Solyc03g121000	Zinc finger CCCH domain-containing protein 4-like	Altered C-terminal sequence
3	Solyc03g121720	Succinic semialdehyde reductase isofom 2	Altered C-terminal sequence
4	**Solyc04g010040**	**RNA recognition motif-containing protein**	Missing most part of the protein, including the RNA recognition motif
4	Solyc04g016350	40S ribosomal protein S4-like isoform 1	Altered C-terminal sequence
5	Solyc05g009260	Transport inhibitor response 1	Altered C-terminal sequence*^b^*
5	Solyc05g013390	Unknown protein	Altered C-terminal sequence
5	Solyc05g017900	EamA-like transporter membrane protein	Altered C-terminal sequence*^a^*
6	Solyc06g005080	Vacuolar protein sorting-associated protein 18	Altered N-terminal sequence*^a^*
6	Solyc06g005450	NAD-specific glutamate dehydrogenase	Altered C-terminal sequence*^b^*
6	Solyc06g065440	C2H2-type zinc finger family protein	Altered C-terminal sequence*^a^*
6	Solyc06g066210	Unknown protein	Altered C-terminal sequence
6	Solyc06g066570	Peroxisome biogenesis protein 2-like	Altered C-terminal sequence
6	Solyc06g084160	Serine/threonine-protein kinase BUD32 homolog	Altered N-terminal sequence*^a^*
7	Solyc07g039330	WD-40 repeat-containing protein MSI4-like	Missing most part of the protein, the WD40 repeat is truncated*^b^*
8	Solyc08g006070	AIG2-like protein-like	Altered C-terminal sequence
9	**Solyc09g018670**	**Transmembrane protein 56-B-like isoform 2**	Missing most part of the protein, the TLC domain is truncated
9	Solyc09g082630	1,2-dihydroxy-3-keto-5-methylthiopentene dioxygenase 1	Altered N-terminal sequence
9	**Solyc09g089690**	**1-aminocyclopropane-1-carboxylate oxidase**	Missing most part of the protein, including the prolyl 4-hydroxylase a subunit
10	Solyc10g037910	Shaggy-related protein kinase eta	Altered C-terminal sequence*^b^*
10	Solyc10g050080	Serine/threonine-protein kinase C01C4.3-like	Missing most part of the protein*^b^*
11	Solyc11g005420	Unknown protein	Altered N-terminal sequence*^a^*
11	Solyc11g006510	Nuclear transport factor 2 (NTF2) family protein	Missing most part of the protein*^b^*
11	**Solyc11g012050**	**Serine/threonine-protein kinase HT1-like**	Missing most part of the protein, including the PTK catalytic domain
11	Solyc11g056680	DNA-damage-repair/toleration protein DRT100-like	Altered C-terminal sequence
11	**Solyc11g067080**	**Protein kinase G11A-like**	Missing most part of the protein, including the STK catalytic domain
12	Solyc12g041980	Breast cancer susceptibility 1 homolog	Altered C-terminal sequence*^b^*
12	Solyc12g055850	Lecithin retinol acyltransferase	Altered C-terminal sequence*^a^*

The table explains the effects on protein modification. Altered N- or C-terminal category marks the proteins up to ~25% of changes on the protein ends. The proteins that are likely to result from wrong predictions in Heinz 1706 (*^a^*), and partial gene sequences (*^b^*) are marked. Important genes possessing large effects are in bold.

Despite the measurable RNA expression levels, a few of the L5 genes containing FS InDels appeared to encode incomplete proteins both in the reference genome and in L5, *e.g.*, *Solyc01g017050* or *Solyc01g091150* (Table 2), based on comparisons with orthologous proteins; this decreases the likelihood of their functional requirement in tomato, so these genes were also considered to be of less interest in our functional analysis. After eliminating these uncertain FS InDel changes still 7 genes exhibit altered protein sequence in L5 (Table 2). These FSs are undoubtedly changing the protein structures as entire domains, motifs are absent (Table S6).

The final group of FS InDel genes to consider are those that affected protein structure similarly in both in E9 and L5 (Table 3). By searching for homologous genes we could divide these FS InDels in two categories: the ones that are mutated from the normal protein structure in both E9 and L5, and, second, the others that appear to be mutated in Heinz 1706 but not in E9 or L5 (Table S7).

**Table 3:** The frame shift altered proteins where E9 and L5 are similar and they both differ from the reference genome

Chr	Gene Number	Gene Annotation	Sequence and Structural Changes Both in E9 and L5 or in Heinz 1706
2	**Solyc02g085420**	**U1 small nuclear ribonucleoprotein 70 kDa**	Missing most part of the protein, including the RNA recognition motif both in E9 and L5
5	Solyc05g051870	Pollen olee1-like protein	Altered C-terminal sequence in Heinz 1706
5	**Solyc05g054640**	**2-oxoglutarate dehydrogenase E1 component**	Missing most part of the protein, including the thiamine pyrophosphate and pyrimidine binding domain both in E9 and L5
5	Solyc05g055680	Putative adenosylhomocysteinase	Missing most part of the protein both in E9 and L5
5	Solyc05g055990	Aquaporin PIP2-7-like	Altered C-terminal sequence in Heinz 1706
6	Solyc06g005210	Cytochrome P450 like	Missing most part of the protein both in E9 and L5*^b^*
7	Solyc07g062310	Plant protein of unknown function,	Altered N-terminal sequence in Heinz 1706*^a^*
		DUF641 domain	
7	**Solyc07g065220**	**Calcium-dependent lipid-binding family protein**	Missing most part of the protein in Heinz 1706
7	**Solyc07g065630**	**E3 ubiquitin-protein ligase UPL1-like**	Missing most part of the protein, including the catalytic domain and E2 ubiquitin-conjugating enzyme interaction site both in E9 and L5
9	**Solyc09g005580**	**Selenoprotein-T protein-like**	Missing most part of the protein including the RDX domain in Heinz 1706
9	**Solyc09g007770**	**Aquaporin PIP2-1-like**	Altered N-terminal sequence both in E9 and L5
10	**Solyc10g083190**	**Myosin-9-like isoform X3**	Altered C-terminal sequence both in E9 and L5
10	**Solyc10g083870**	**Tankyrase-1-like**	Missing most part of the protein, including the ankyrin repeat both in E9 and L5
12	Solyc12g010020	Leucine aminopeptidase	Altered C-terminal sequence in Heinz 1706
12	**Solyc12g011030**	**Xyloglucan endotransglucosylase-hydrolase XTH9 precursor**	Altered internal (exonal) sequence in Heinz 1706

The table explains the effects of the frame shift on protein structure. Altered N- or C-terminal category indicates that the proteins are up to ~25% different at one of the termini. The proteins likely to have a bad annotated prediction in Heinz 1706 SL2.40 reference (*^a^*) or a partial gene sequence (*^b^*) are marked. Important genes possessing large effects are indicated in bold.

### Functions of genes containing disruptive InDels in E9 and L5

To verify the specificity of the determined nine FS InDels responsible for significantly altered proteins in these lines, we next made a comparison with another 12 related cultivars and wild species from the list of 150 tomato accessions which have been re-sequenced (100 Tomato Genome Sequencing Consortium *et al.* 2014). As expected, most of the revealed InDels are not uniquely present in the E9 and L5 lines, but were represented within a limited number of other cultivars (Table 4). As the E9 and L5 derived P population was generated to localize QTL responsible for salt tolerance in stress conditions we were interested whether any of the putatively influential InDels could somehow be involved in regulation of abiotic stresses responses. The E9-disrupted gene Solyc03g115650, a translation initiation factor 5A-1, is a stress-responsive protein involved in the abscisic acid (ABA) signal transduction pathway in *Tamarix androssowii*, a tropical plant possessing high drought and salt tolerance (Wang *et al.* 2012). Beside E9 line, this gene has the same FS InDel in *S. lycopersicum* cv “Rutgers” (SLR), *S. lycopersicum* cv “Katinka Cherry” (SLKC) and *S. lycopersicum* cv “Black Cherry” (SLBC) lines (Table 4), from which the last two are closely related cherry tomato cultivars. The *Solyc12g038920* gene, having FS deletion, codes for a serine-threonine kinase and shows high homology for a rice C-type cyclin-dependent protein kinase, which is induced by salt stress and ABA treatments, probably involved in the plant developmental program and ABA-signaling pathway (Huang *et al.* 2008). This FS seems to be very specific for the E9 line as it is not present in any other line (Table 4). The E9 gene Solyc12g100290 containing an FS InDel encodes a histone-lysine N-methyltransferase homolog, which is related to the *SUVH* gene family in *Arabidopsis thaliana* (Thorstensen *et al.* 2011). This family is responsible for epigenetic silencing of chromatin histones and participates in the regulation of genes and transposons, which can be induced by biotic or abiotic stresses. This mutation is present also in the heritage cultivar *S. lycopersicum* cv Ailsa Craig (SLAC) and the SLKC line.

**Table 4:** The occurrence of selected E9 and L5 frame shift alterations in other tomato accessions

	E9	L5	*S. pim.* LYC2798	*S. pim.* LA1584	*S. pim.* LA1578	*S. lyc. cv* Moneymaker	*S. lyc. cv* Ailsa Craig	*S. lyc. cv* Rutgers	*S. lyc. cv* Black Cherry	*S. lyc. cv* Katinka Cherry	*S. lyc. cerasi.* Plovdiv INDEL	*S. lyc. cerasi.* Levovil INDEL	*S. lyc. cerasi.* Cervil INDEL	*S. lyc. cerasi.* LA0147 INDEL
Solyc03g115650	**+**							**+**	**+**	**+**				
Solyc12g038920	**+**													
Solyc12g100290	**+**						**+**			**+**				
Solyc01g090610		**+**	**+**											
Solyc03g098240		**+**	**+**											
Solyc04g010040		**+**	**+**						**+**	**+**			**+**	
Solyc09g018670		**+**	**+**										**+**	
Solyc09g089690		**+**	**+**							**+**			**+**	
Solyc11g012050		**+**	**+**		**+**									
Solyc11g067080		**+**	**+**	**+**	**+**	**+**	**+**	**+**	**+**	**+**			**+**	
Solyc02g085420	**+**	**+**						**+**		**+**				
Solyc05g054640	**+**	**+**	**+**	**+**	**+**	**+**	**+**	**+**	**+**	**+**			**+**	**+**
Solyc07g065220	**+**	**+**	**+**	**+**	**+**	**+**	**+**	**+**	**+**	**+**				
Solyc07g065630	**+**	**+**	**+**	**+**	**+**	**+**	**+**	**+**	**+**	**+**	**+**			
Solyc09g005580	**+**	**+**	**+**	**+**	**+**	**+**	**+**	**+**	**+**	**+**		**+**	**+**	**+**
Solyc09g007770	**+**	**+**	**+**	**+**	**+**		**+**	**+**	**+**	**+**			**+**	**+**
Solyc10g083190	**+**	**+**	**+**	**+**	**+**	**+**	**+**	**+**	**+**	**+**	**+**			
Solyc10g083870	**+**	**+**	**+**	**+**	**+**	**+**	**+**	**+**	**+**	**+**	**+**			
Solyc12g011030	**+**	**+**	**+**	**+**	**+**	**+**	**+**	**+**	**+**	**+**	**+**	**+**	**+**	**+**

The table shows a nonexhaustive list of the tomato accessions from the 150 tomato genome resequencing project that have the same FS InDels as E9 and/or L5.

Our FS InDel data on L5 also was investigated in other *S. pimpinellifolium* sequences. The three available lines from the 150 tomato genome resequencing project (LYC2798, LA1584, LA1578) have also these InDels; however, only the LYC2798 line possess the same InDel distribution as L5 (probably a very close line), whereas LA1584 and LA1578 have fewer of the investigated InDels (Table 4). *Solyc01g090610* codes for a gibberellin 2-beta-dioxygenase homolog which is induced by salinity stress in rice (Mizuno *et al.* 2010); this enzyme is responsible for inactivating gibberellins. Solyc03g098240 is a glutamate decarboxylase homolog, which catalyses the conversion of glutamate into gamma-aminobutyric acid. Exogenous gamma-aminobutyric acid treatment protects tomato seedlings from chilling stress by enhancing antioxidant enzymes activities and maintaining membrane integrity (Malekzadeh *et al.* 2014). These two genes have exclusively changed in L5 and LYC2798 line. The *Solyc04g010040* gene codes for an RNA recognition motif-containing protein and belongs to the large RNA binding protein family whose members are known to be involved in regulatory processes in response to different abiotic stress conditions (Ambrosone *et al.* 2012). Interestingly, while the other *S. pimpinellifolium* lines LA1584 and LA1578 have no InDel in *Solyc04g010040*, the SLBC and SLKC tomato lines do. Moreover, another cherry line, *S. lycopersicum* var. *cerasiforme* “Cervil” (SLCC) also has this InDel change. This tomato variant possesses similarities with L5 as the SLCC line is an admixture of *S. lycopersicum* and *S. pimpinellifolium* lines (Causse *et al.* 2013). Solyc09g089690 is an aminocyclopropane-1-carboxylate oxidase protein, which is induced in tomato by attack of a certain *Fusarium oxysporum f.sp. radicis-lycopersici* (Çakir *et al.* 2014) and is an enzyme in ethylene biosynthesis. The same InDel is present in SLKC and SLCC lines. Solyc11g012050 is a HT1-like serine-threonine-protein kinase, which regulates the stomatal closure in *A. thaliana* leaves via the ABA-signaling pathway (Hashimoto *et al.* 2006). The FS InDel within this gene is only present in *S. pimpinellifolium* lines L5, LYC2798 and LA1578. Solyc11g067080 is a protein kinase which shows high homology to kinesin-like calmodulin-binding proteins that are primarily responsible for the microtubule-based movement of the plant cell (Vinogradova *et al.* 2013). Nonetheless, this FS InDel seems to be less specific as it can be found in several other lines including *S. lycopersicum* cv “Moneymaker,” SLAC, SLR, SLKC, and SLBC tomato as well. The function of Solyc09g018670, a transmembrane protein 56B is not yet analyzed in plants, however, it seems to possess a highly homologous, conservative sequence in *A. thaliana* and soybean. The FS InDel of this gene is also present in the SLCC admixture line.

The FS InDels of E9 and L5 show rather specific presence in a few lines. Interestingly, most of them are present in SLKC and SLCC lines suggesting common progenitors between them. The function of most genes were directly related to abiotic stresses, including salt tolerance properties, which are the target traits in the E9 × L5 RIL population.

### Important InDels that occur in both E9 and L5

From the significant FS InDels, by homology searches we concluded that the *Solyc02g085420*, *Solyc05g054640*, *Solyc07g065630*, *Solyc09g007770*, *Solyc10g083190* and *Solyc10g083870* genes have sequence alteration both in E9 and L5 compared to the functional one in Heinz 1706 (Table S7). Although, in these cases the non-functional FS InDels seems to be not limited only to the two parental lines, most investigated cultivars carry these InDels, which suggests that the functions of the affected genes are likely less significant and not critical. Only the gene of the U1 small nuclear ribonucleoprotein 70 (Solyc02g085420), a member of the spliceosomal machinery (Valadkhan and Jaladat 2010) seems to have the FS only in limited number of lines, in SLR and SLKC in addition to E9 and L5 (Table 4).

On the other hand, the FS InDels genes of *Solyc07g065220*, *Solyc09g005580* show truncated and probably non-functional gene sequence in Heinz 1706. These FS changes seem to be rather specific for the reference genome as most other investigated lines have the obviously correct gene sequence (Table 4). Solyc07g065220 is a calcium-dependent lipid-binding protein that shows high homology to the C2-domain containing phospholipid-binding protein of rice, which is required for pollen fertility (Yang *et al.* 2008). The function of Solyc09g005580 orthologs is described only in the animal kingdom so far. The Heinz 1706 *Solyc12g011030* gene carries a different exon sequence than the rest of the lines, however, both types can be functional. All these InDel data may be highly useful during possible molecular analyses of these particular genes, or as candidate genes in QTL mapping.

Next-generation sequencing−based resequencing greatly facilitates investigations in plant genetics, speeding up genetic mapping projects and breeding programs. In our study, we used deep sequencing data of the two parental lines to discover SNPs and InDels for use in the fine-mapping of QTL in the descendant RIL populations. The sequence data are now publically available in the interactive and decipherable Genoverse format to search for essential SNPs and InDels which can be particularly useful for studies related to the *S. pimpinellifolium* genome. We have revealed regions which may harbor new introgressions in both E9 and L5 and determined which InDels are most likely to strongly influence gene functions. The data generated have particular importance for genetic analysis: several salinity and rootstock related QTL of the E9 and L5 derived P population are under investigation in different laboratories where these data will be particularly valuable. The detailed SNP and InDel data provide very convenient PCR markers for fine mapping, the InDel functional analysis provides many genes for further functional investigation, particularly where co-localization with QTL is observed. The FS InDel changes that we characterized undoubtedly represent only a small portion of the numerous probable gene function changes, but our investigation of this class of mutation underlined the limitations of existing algorithms which generated many pseudo FS InDels.
